# Semen Characteristics of Purebred and Crossbred Male Rabbits

**DOI:** 10.1371/journal.pone.0128435

**Published:** 2015-05-28

**Authors:** Mahmoud Salah El-Tarabany, Khairy El-Bayomi, Tamer Abdelhamid

**Affiliations:** Animal Wealth Development Department, Zagazig University, Sharkia, Egypt; Universidad Nacional Autonoma de Mexico, MEXICO

## Abstract

The aim of this investigation was to evaluate the semen quality traits of purebred male rabbits and their crosses under subtropical Egyptian conditions. A full 3 x 3 diallel crossing was performed for producing the first generation progeny of New Zealand White (N), Flander (F) and Rex (R) breeds. The highest ejaculate volume (p< 0.05) and percentage of live sperms (p<0.01) with the lowest percentage of sperm cell morphological abnormalities (p<0.05) had been recorded in the NF bucks. Moreover, they possessed positive estimates of direct heterosis for ejaculate volume, mass motility (Mm), individual motility (Im) and sperm cell concentration (SCC). On the contrary, pH had negative estimates of direct heterosis in all crosses and their reciprocal. Semen pH was negatively correlated with SCC (r = -0.18), Mm (r = -0.13) and Im (r = -0.23). In conclusion, the superiority of crossbreeding was particularly obvious in the New Zealand White x Flander males, which cumulated heterosis and favorable maternal effects of the Flander dams.

## Introduction

Undoubtedly bucks are the basis of the reproductive success in the rabbit farms, but they have not received the attention they should have, mainly if we consider that single male is affecting the prolificacy and fertility of many does particularly when the artificial insemination (AI) is performed as a routine in the rabbit farm [1]. The objective of the AI centers is to obtain a greater number of doses at lower cost, and at the same time it should allow semen production to be increased while maintaining a high level of male fertility and prolificacy. The success of rabbits AI program depends to a great extent on male health and reproductive performance at the time of semen collection; lack of seasonal impacts on libido, producing large volume of sperm, sperm motility and viability with a few numbers of abnormalities. In rabbits, optimal reproductive results are achieved with commercial AI largely because the procedures aim to maximize the probability of oocytes being fertilized; however characteristics of semen are highly variable between collections [2]. The amount of produced semen and its quality, play a crucial role in bucks fertility and prolificacy [3,4]. The number of inseminating doses generated from each ejaculate depends on the volume, sperm concentration, and motile sperm after freezing/thawing. Also, techniques of semen preservation (fresh, refrigerated and frozen) and insemination (vaginal, cervical and intrauterine) are considered as very important techniques in genetic improvement programs [5]. Semen quantity and quality is commonly described by means of a wide domain of traits, including qualitative traits of the ejaculate, characteristics associated with the biochemical composition of the ejaculate, qualitative characteristics of the spermatozoa and characteristics of the inseminating dose [6,7]. The capability of AI in animal breeding implies decreasing the number of spermatozoa at insemination. An assessment of semen quality is therefore desired. In rabbits, most of the studies regarding the relationship between fertility and semen quality have only considered sperm concentration or sperm motility as the major parameters that indirectly define semen quality [8,9].

Differences may occur between purebred and crossbred males with regard to reproductive traits; however, there is a very little information available concerning the use of crossbred male rabbits. Mating involved both F_1_ crossbred males and females had higher fertility and litter size, when compared to mating purebred [10]. The magnitude of heterosis for semen traits might encourages the use of crossbred bucks in the semen production farms. The estimated values of heterosis in INRA1601 x NRA2066 crossbred rabbit strains were significant and relevant for concentration, mass motility and the percentage of motile sperm per ejaculate [11,12]. Therefore, the aim of the present study was to evaluate the semen quality parameters of purebred and crossbred male rabbits under subtropical Egyptian conditions.

## Materials and Methods

This work was reviewed and approved by the Animal Care and Welfare Committee of Zagzaig University, Egypt (ANWD-215). It was conducted at the Experimental Building Unit, Faculty of Veterinary Medicine, Zagazig University.

### Animals and management

The experimental flock consisted of three rabbit breeds, New Zealand white (N), Rex (R) and Flander (F). Concerning each parent breed, fifteen does and six bucks were used to obtain the first generation purebred and crossbred animals. Bucks and does have been selected at the 6^th^month of age. Breeding animals were kept individually in separate wire cages (40 x 60 x 50 cm W x Lx H), supplied with manual feeders and nipple system for watering. Metal nest box was attached to the doe's cage (40 x 40 x 40 cm). Rabbits were fed a commercial pelleted diet *ad-libitum*, during the whole experimental period. The diet composition on a dry- matter basis contains 18.5% crude protein, 8% crude fiber, 3.0% ether extract and 6.5% ash. Throughout the period of experiment, bucks were exposed to subtropical conditions, where the average minimum temperature ranged from 21.9 to 23.8°C, and average maximum temperature was 32.1 to 34.3°C. Good ventilation and fresh air were provided to minimize ammonia level in the house, as well as hygienic elimination of wastes. Fourteen hours of daily light were provided. Mature animals were mated in a full 3 x 3 diallel cross design. Accordingly, each doe was naturally mated with her assigned buck, where no visual or physical contact allowed within the house. However, no synchronization protocols were conducted in the breeding does. This diallel cross design resulted in 3 purebred lines (NN, FF and RR), and 6 crossbred lines (NR, NF, RF, RN, FN and FR; with the male mentioned the first).54 Doe, buck number and date of mating were registered, and then each doe was palpated for pregnancy 14 days after mating. The expected date of kindling was recorded for positively palpated does, while negatively palpated does were remated immediately.

Just after kindling, examination of the first generation litters were carried out and dead kits were removed. Kits were weaned, ear tagged and separated in cages at the 4^th^week of age. They were reared under the same managerial and nutritional conditions, where individual records were founded for each breeding animal. At three months of age, young rabbits were sexed. Concerning each genetic group, twenty males (purebreds, crossbreds and reciprocal) were picked randomly, kept under the same managerial and nutritional conditions, housed in individual cages to complete the growing period till 5 months of age (ready for semen collection).

### Semen collection and examination procedures

Bucks started the training period at the 5^th^month of age, where the experimental breeds reached the sexual maturity. Over a period of 4 weeks, only one ejaculate was collected per male weekly, using an artificial vagina [13]. Bucks have been subjected to the experimental evaluation at the 6^th^ month of age. Two ejaculates were collected per male weekly, with an interval of 30 minutes between collections for 6 consecutive weeks [14].

Concerning each genetic type, 240 ejaculates were collected, stocked at 37°C in a water bath, and then evaluated within 15 minutes after collection. Ejaculates comprising urine and calcium carbonate deposits were neglected. Gel plugs, when present, were removed before the volume of the ejaculates determined using a graduated tube. The pH cooperative paper ranging from 0 to 14 with one grade was used to calibrate the pH value. Mass motility (Mm) was estimated according to a subjective scale ranging from 1 to 9 [15], using aliquots (10 μl) of raw semen and a light (Nikon) microscope at magnification 10x.Also aliquots (10 μl) of raw semen were diluted with one drop of 2.9% sodium citrate and inspected under light (Nikon) microscope at magnification 40xforthe evaluation of individual motility (Im %). Aliquots (10 μl) of raw semen were fixed using a vital nigrosin-eosin staining to allow later measurements of semen quality traits by examining 200 spermatozoa under a light (Nikon) microscope at magnification 1000xand calculating the percentages of live spermatozoa, dead spermatozoa and sperm cell morphological abnormalities[16]. Sperm cell concentration (x 10^6^/ml) was quantified by direct cell count using the improved Neubauer haemocytometer in aliquots at magnification 400x [17].

### Statistical analysis

Semen quality traits were analyzed using General Linear Model (PROC GLM) procedure of the SAS statistical system Package V9.1 [18]. The model included the following effects:

$$Y_{ijkl}=\mu +G_{i}+R_{j}+S_{k}+e_{ijkl}$$

Where *Y*_*ijkl*_ represents the value of semen quality traits (volume, pH, Mm, Im, SCC and sperm cell morphological abnormalities) measured in the *l* ^th^ animal; *μ* is the overall mean for each trait; *G*_*i*_ is the fixed effect of *i* ^th^ genetic types with 9 levels (*i* = NN, FF, RR, NR, NF, RF, RN, FN and FR); *R*_*j*_ is the fixed effect of ejaculate rank with two levels (j = 1^st^ and 2^nd^), *S*_*k*_ is the fixed effect of semen Batch and *e*_*ijkl*_ is the random residual effect. The data are reported as least square means and residual standard deviations (RSD; square root of the mean square errors). P-values of <0.05 were considered to be statistically significant. Pearson’s correlation test was applied to determine the phenotypic correlation among semen quality traits. Direct heterosis (H^d^) has been estimated as a difference between the average of crossbred and purebred males and expressed as a percentage of the paternal average according to the genetic model of Dickerson [19].

## results

### Purebred versus crossbred effects on semen characteristics

Significant differences (P<0.05) were observed among different genetic types (purebred, crossbred and reciprocal) for the most of semen quality traits including volume, percentage of live spermatozoa and sperm cell morphological abnormalities (Table 1). The NF males recorded the highest ejaculate volume (0.64 ml) and the superior percentage of live sperms (95.13%). Otherwise, the opposite is true in the reciprocal FN crossbred males. The NF males had the lowest values for head abnormalities (HA) %, neck—midpiece abnormalities (NMA) %, tail abnormalities (TA) %, proximal cytoplasmic droplets (PD) % and distal cytoplasmic droplets (DD) %. On the contrary, the FR reciprocal crossbred males had higher scores for HA %, TA %, PD % and DD %.

**Table 1:** Semen quality traits and sperm cell morphological abnormalities (%) for the first generation of purebred and crossbred male rabbits.

Trait	Genetic type									^1^RSD	P-value	^2^SB	^3^ER
	NN	FF	RR	NR	NF	RF	RN	FN	FR				
Volume	0.52^a^ ^b^	0.45^c^	0.57^a^ ^b^	0.42^c^4	0.64^a^	0.48^b^ ^c^	0.47^b^ ^c^	0.43^c^	0.49^b^ ^c^	0.11	0.022	0.652	0.311
pH	7.56^b^	7.65^a^ ^b^	7.87^a^	7.62^b^	7.57^b^	7.55^b^	7.62^b^	7.61^b^	7.57^b^	0.40	0.041	0.764	0.186
Mm	7.59^a^ ^b^	7.53^a^ ^b^	7.28^b^ ^c^	7.56^a^ ^b^	7.46^a^ ^b^	7.86^a^	7.40^a^ ^b^	7.39^a^ ^b^	7.39^a^ ^b^	0.76	0.003	0.322	0.024
Im	94.1^a^ ^b^	94.6^a^ ^b^ ^c^	92.0^b^ ^c^	92.8^a^ ^b^ ^c^	95.3^a^ ^b^	93.4^a^ ^b^ ^c^	93.4^a^ ^b^ ^c^	96.1^a^	91.2^c^	5.86	0.024	0.030	0.048
SCC	737	660	511	585	709	702	673	683	698	89.75	0.092	0.022	0.593
LS	94.1^a^ ^b^	92.2^b^ ^c^	92.6^a^ ^b^ ^c^	93.1^a^ ^b^ ^c^	95.1^a^	93.4^a^ ^b^ ^c^	91.5^b^ ^c^	90.6^c^	92.1^b^ ^c^	7.18	0.006	0.334	0.263
HA	3.95^c^	5.69^b^	4.96^c^	4.88^b^ ^c^	2.50^d^	5.40^b^ ^c^	4.96^b^ ^c^	5.56^b^	7.13^a^	1.46	0.022	0.731	0.194
NMA	4.90^c^	5.73^b^ ^c^	5.56^b^ ^c^	5.36^b^ ^c^	2.93^d^	5.09^c^	5.55^b^ ^c^	7.13^a^	6.52^a^ ^b^	1.18	0.003	0.266	0.031
TA	3.09^c^	7.46^a^ ^b^	4.76^a^ ^b^	4.44^a^ ^b^	2.80^c^	3.72^c^ ^b^	4.88^a^ ^b^	4.56^a^ ^b^	5.60^a^	1.11	0.012	0.041	0.456
PD	1.50^a^ ^b^ ^c^	1.69^a^ ^b^	1.52^a^ ^b^ ^c^	1.12^b^ ^c^	0.66^c^	1.59^a^ ^b^	1.40^a^ ^b^ ^c^	1.65^a^ ^b^	2.13^a^	0.88	0.005	0.013	0.177
DD	1.18^a^	1.26^a^	1.20^a^	1.24^a^	0.36^b^	1.59^a^	1.33^a^	1.56^a^	1.91^a^	1.33	0.044	0.454	0.382

N: New Zealand White, F: Flander, R: Rex, with the male mentioned the first

Mm: Mass motility, Im: Individual motility, SCC: Sperm cell concentration (x 10^6^ / ml), LS: live sperm, HA: Head abnormalities, NMA: Neck—midpiece abnormalities, TA: Tail abnormalities, PD: Proximal cytoplasmic droplets, DD: Distal cytoplasmic droplets.

^1^ Residual standard deviation

^2^Semen batch (*p*-value)

^3^ Ejaculate rank (*p*-value)

^a,b,c^Means within the same row having different superscripts are significantly differed.

### Phenotypic correlation among different semen quality parameters

Individual motility (Im) had a significant (p< 0.01) positive correlation with SCC (r = 0.16; Table 2). Also, percentage of live sperm was positively correlated with the percentage of abnormal sperms (r = 0.12). On the contrary, Im had a significant (p< 0.05) negative correlation with the percentage of abnormal sperms (r = - 0.12). Semen pH was negatively correlated with SCC, Mm and the percentage of motile spermatozoa (r = - 0.18,- 0.13 and—0.23, respectively).

**Table 2:** Phenotypic correlations among semen quality parameters.

Trait	pH	Mm	Im %	SCC	LS	AS
Volume	- 0.08	0.03	- 0.01	- 0.05	0.11	0.11
pH		- 0.13^b^	-0.23^a^	- 0.18^a^	- 0.12^b^	0.03
Mm			0.04	0.04	0.04	0.05
Im %				0.16^a^	0.05	- 0.12^b^
S.C.C					0.06	- 0.05
LS						0.12^b^

Mm: Mass motility, Im: Individual motility, SCC: Sperm cell concentration, LS: live sperm, AS: abnormal sperm.

^**a**^Significant at the level (P < 0.01).

^**b**^ Significant at the level (P < 0.05).

### Heterosis percentages

All crosses and their reciprocal revealed positive estimates of heterosis for Mm, but they were negative for pH and PD % (Table 3). The NF crossbred bucks had positive estimates of direct heterosis for volume of ejaculate, Im, SCC and the percentage of live sperms. Also, they had favorable negative estimates for all sperm cell morphological abnormalities, while the opposite trend was observed in their reciprocal crossbred males (FN).

**Table 3:** Heterosis percentages of semen quality traits for the crossbred male rabbits

Trait	Genetic type		
	H^d^_NR_	H^d^_NF_	H^d^_RF_
Volume	-13.40^b^	17.43^b^	-5.88^a^
pH	-0.52	-1.81	-2.7^a^
Mm	0.26	0.54	6.21^a^
Im	-1.69	2.43^a^	0.16
SCC	-16.18^b^	13.59^a^	19.85^a^
LS	-.0.03	1.84^a^	1.06
HA	65.16^a^	-43.82^b^	57.61^a^
NMA	36.32^b^	-43.98^b^	24.44^a^
TA	42.59^a^	-28.75^a^	6.49
PD	-51.20^a^	-56.29^a^	-27.08^b^
DD	2.94	-69.74^a^	42.85^a^

H^d^: direct heterosis.

Mm: Mass motility, Im: Individual motility, SCC: Sperm cell concentration, LS: live sperm, HA: Head abnormalities, NMA: Neck—midpiece abnormalities, TA: Tail abnormalities, PD: Proximal cytoplasmic droplets, DD: Distal cytoplasmic droplets

^**a**^ Significant at the level (P < 0.05)

^**b**^ Significant at the level (P < 0.01).

## Discussion

The primary objectives of this study were to evaluate the semen quality parameters of purebred (N, F and R) and their crossbred male rabbits in a complete 3 x 3 diallel crossbreeding design. An improvement in the production of potentially fertile doses could be achieved through the use of crossbreed males, in consequence of positive heterosis and complementarity between parental lines [20]. The current study demonstrated that New Zealand White x Flander hybrid produces the most voluminous ejaculates; however, the relatively low ejaculate volume in different genetic types might be due to practice that semen was collected from those bucks just after maturation. Similarly, this notation has been reported in some earlier studies which were conducted on young mature bucks [21]. Amelioration of the semen quality parameters in crossbred males was expected and could be useful particularly if one likes to use the semen of crossbred bucks on a major scale in AI programs. These improvements accomplished in some crossbred bucks of the current study could be explained on the basis that crossbreeding influences positively growth rate of the whole body. This leads to an early maturation of the hypothalamus and the pituitary, which directly manipulates the growth of testes and finally the performance of the bucks [22]. There was a positive genetic correlation (0.36) between the volume of ejaculate and body gain in paternal lines of rabbits selected for growth rate [23]. Also, a favorable genetic correlation (0.21) has been detected between daily gain and sperm concentration [2]. In chickens, body gain was positively correlated with ejaculate volume, pH and abnormal sperm [24]. The higher ejaculate volume in the NF males than that in some purebred and crossbred lines could be attributed to their superior growth rate and suspected early maturity of their pituitaries in comparison with other lines [2]. This can positively affect the secretion of lutinizing hormone (LH) which affects the secretion of testosterone from the interstitial tissues of the testes. The testosterone might not reach to the required level for full stimulation of the accessory sex gland and the epididymis that influence the volume and the maturity of ejaculate [25]. Decreased testosterone concentration had resulted in a reduced volume of testis and prostate. Furthermore, spermatogenesis has been arrested with development not occurring beyond the spermatogonia or spermatocyte stages [26]. Venge and Frölich [27] stated that the differences in volumes between the studied genetic types (Polish—a small body sized breed, two large sized breeds and one crossbred population) might be attributed to the weight of bucks, but the differences in concentration did not follow this. The crossbred males were 1.1–2 times higher in the number of delivered sperms than that in pure breeds and this may be due to the effect of heterosis. Furthermore, body weight of male rabbits significantly correlated with semen characteristics, reflecting the importance of physical fitness for semen quantity and quality [28]. This characteristic is economically critical, as it is used to determine the number of inseminating doses prepared from an ejaculate. On the contrary, Abo El-Ezz et al. [29] compared imported purebred bucks (Chinchilla and Bouscat) with the reciprocal crossbreds of these breeds and two local Egyptian strains. They did not detect any crossbred superiority for the volume and concentration over the imported breeds; however, crossbred bucks had lower percentage of dead and abnormal spermatozoa. Additionally, there were some hybrid boars that produced ejaculates with low quality and quantity than the purebred animals [30]. Sperm motility is a very important parameter that reflects the quality and has a significant effect on egg cell fertilization [31]. Moreover, there was a positive correlation between sperm motility and the number of piglets delivered at birth [32]. Additionally, ejaculates of purebred males in the present work contained spermatozoa with symmetrical motility in comparison with ejaculates of hybrid bucks.

The relatively high percentages of cytoplasmic droplets in different genetic lines might be attributed to the fact that semen collection was performed just after maturation of males [33]. Similar results had been recorded in a crossbreeding trial involving Saudi Gabalai with V-Line rabbits [25]. They reported a significant improvement in semen parameters of crossbred bucks (sperm concentration, abnormal sperms and dead sperms) than in purebred. In the current study, semen pH was negatively correlated with concentration, mass motility and the percentage of motile spermatozoa. An opposition had been reported previously between pH and other sperm traits, particularly concentration and mass motility [34,35]. This may be attributed to the metabolic activity of the spermatozoa, which use fructose as the major source of energy and release lactic acid that decreases pH [36,37]. Similar studies detected a positive correlation between the volume of ejaculate and mass motility, while the volume of ejaculate and SCC was negatively correlated [38]. Brun et al. [11] reported that, pH was negatively correlated with the percentage of motile spermatozoa (r = - 0.13), concentration (r = - 0.30) and mass motility (r = - 0.16).

The NF bucks recorded superior direct heterosis estimate for the percentage of live sperms, with favorable negative estimates for the percentages of all sperm cell morphological abnormalities. One of the explanations for positive heterotic effects in the percentage of sperm motility could be due to faster sexual maturation in crossbred bucks than that in purebred [25]. Regarding the comparison between reciprocal crosses, Flander would exert favorable maternal effects on these traits compared to others. Maternal effects are indeed only one of the possible explanations for differences between reciprocal crosses; sex-linked or imprinting effects might also explain such differences. Shrestha and Fahmy [39] recorded favorable estimates of maternal heterosis, represented in the use of crossbred bucks characterized by high volume of ejaculate, high semen quality with more concentration and motile sperms, along with low percentages of abnormal and dead sperms. The effect of the mitochondria, maternally transmitted cell organites involved in energy metabolism, is a particularly appealing hypothesis for traits related to metabolism such as mass motility or percentage of motile sperm [11]. Our findings confirmed those obtained previously [40]. Not only they estimated positive direct heterosis for mass motility in all crosses of New Zealand White, Californian and Rex, but also they estimated negative heterosis for the percentage of dead sperms and ejaculate volume. Our estimates of direct heterosis for sperm concentration, ejaculate volume and percentage of live spermatozoa were compatible with the others researchers [41]. Moreover, they found a favorable direct heterosis effect for ejaculate volume (10.6%), sperm concentration (13.6%), sperm motility (10.5%) and for percentage of abnormally shaped and dead spermatozoa (-21.5% and-20.3%, respectively) in the cross scheme of a Spanish maternal line and a Saudi breed performed to achieve 2 new synthetic maternal lines. Brun et al. [11] reported high variability in the estimates of heterosis in function of the seminal trait since they observed 6.8% heterosis for mass motility, 4.1% for percentage of motile sperms, 37.5% for SCC, 37.6% for total number of spermatozoa per ejaculate and 42.3% for the number of motile sperms per ejaculate. Additionally, Smital et al. [42] found positive and prominent effects of heterosis in Hampshire × Pietrain crosses on ejaculate volume (30.6%) and ejaculate sperm count (18.24%) in boars. On the contrary, high variability in the estimates of direct heterosis for several semen characteristics were documented, being very high positive for the percentage of spermatozoa with presence of proximal cytoplasmatic droplet [14, 43].

In conclusion, heterotic effects had been evidenced for the most of semen characteristics predominantly the volume, motility, concentration and the percentage of live sperms. Crossbred superiority was mainly evident in the New Zealand White x Flander bucks which cumulated heterosis and favorable maternal effects of the Flander dams. It is possible that the favorable estimates of direct heterosis obtained for most semen parameters may be promoting the rabbit producers to use crossbred bucks on commercial scale. However, further research is needed in order to understand the fertility differences between crossbred and purebred males and the relationship between the fertility of bucks and quantitative seminal characteristics.

## Supporting Information

S1 Appendix General and specific combining ability for different semen characteristics. (DOCX)
