# Supplementary material for: Semen Characteristics of Purebred and Crossbred Male Rabbits
Source: PLoS One. 2015 May 28;10(5):e0128435. doi: 10.1371/journal.pone.0128435 (PMC4447379; doi:10.1371/journal.pone.0128435)
Supplement: S1 Appendix — (DOCX) [file pone.0128435.s001.docx]

**APPENDIX S1**

Semen characteristics of purebred and crossbred male rabbits

Mahmoud Salah El-Tarabany^*^, Khairy El-Bayomi, Tamer Abdelhamid

*Animal Wealth Development Department, Faculty of Veterinary Medicine, Zagazig University,Sharkia, Egypt.*

^*^To whom correspondence should be addressed: Mahmoud Salah El-Tarabany, Department of animal wealth development, Faculty of Veterinary Medicine, Zagazig University, El-Zeraa str. 114; 44511-Zagazig; Egypt

Tel: +00201223668785, Fax: 020552283683

Email: [mahmoudtarabany2887@yahoo.com](mailto:mahmoudtarabany2887@yahoo.com)

mmohamedibrahim@zu.edu.eg

**S1 Appendix. General and specific combining ability for different semen characteristics**

Table 1. General and specific combining ability for semen quality traits in different crosses of New Zealand white, Rex and Flander rabbits

| Trait | Semen quality | | | | | |
| --- | --- | --- | --- | --- | --- | --- |
|  | Volume (ml) | PH | Mm | Im % | S.C.C (×10^6^/ml) | DS |
| GCA | |  |  |  |  |  |
| NN | 0.03 | -0.02 | 0.0048 | 0.62 | -22.88 | 0.44 |
| RR | -0.039 | 0.034 | -0.0028 | -0.76 | -95.30 | 0.37 |
| FF | 0.01 | -0.01 | 0.024 | 0.14 | 118.57 | -0.83 |
| SCA | |  |  |  |  |  |
| NR | -0.065 | -0.12 | 0.05 | 1.65 | 107.49 | -0.73 |
| NF | -0.03 | -0.07 | -0.04 | 3.4 | -39.65 | -0.37 |
| RF | 0.002 | -0.19 | 0.17 | 3.77 | 36.9 | 0.05 |

GCA: general combining ability; SCC: specific combining ability.

Mm: Mass motility, Im: Individual motility, SCC: Sperm cell concentration, DS: dead sperm.

Table 2. General and specific combining ability for Sperm cell abnormalities (%) in different crosses of New Zealand white, Rex and Flander rabbits

| Trait | Live sperms (%) | Sperm cell abnormalities % | | | | |
| --- | --- | --- | --- | --- | --- | --- |
|  |  | HA % | NMA % | TA % | PD % | DD % |
| GCA | |  |  |  |  |  |
| NN | -0.34 | -0.55 | -0.09 | -0.20 | 0.001 | -0.19 |
| RR | -0.23 | 0.34 | -0.05 | 0.16 | -0.05 | 0.28 |
| FF | 0.56 | 0.21 | 0.14 | 0.05 | 0.05 | -0.09 |
| SCA | |  |  |  |  |  |
| NR | 0.72 | 0.68 | 0. 58 | 0.70 | -0.23 | -0.05 |
| NF | 0.39 | -0.07 | -0.03 | -0.16 | -0.69 | 0.03 |
| RF | 0.11 | 1.26 | 0.71 | 0.46 | 0.25 | 0.33 |

GCA: general combining ability; SCC: specific combining ability.

HA: Head abnormalities, NMA: Neck - midpiece abnormalities, TA: Tail abnormalities, PD: Proximal cytoplasmic droplets, DD: Distal cytoplasmic droplets.
